# Identification of key amino acids defining conformational neutralizing epitopes of coxsackievirus A5 using monoclonal antibody escape mutants

**DOI:** 10.3389/fimmu.2026.1837533

**Published:** 2026-04-29

**Authors:** Ke Xu, Jie Pei, Chen Wang, Jing Guo, Xiao-Qi Chen, Shuo Shen

**Affiliations:** 1 Wuhan Institute of Biological Products Co., Ltd., Wuhan, China; 2 Vaccine Technology Innovation Center of Hubei Province, Wuhan, Hubei, China

**Keywords:** antigenic mapping, conformational neutralizing epitopes, coxsackievirus A5, escape mutants, monoclonal antibody

## Abstract

**Background:**

Coxsackievirus A5 (CVA5) is an emerging pathogen associated with severe hand, foot, and mouth disease. Protective antibodies are critical for protection from CVA5 infection, yet the antigenic determinants of CVA5 remain poorly defined. In this study, we aimed to map the conformational neutralizing epitopes of CVA5 using monoclonal antibodies (mAbs) and escape mutant analysis.

**Methods:**

Two IgG, one IgA, and five IgM neutralizing mAbs were purified and characterized for binding affinity and neutralization potency. Antibody pressure selection was applied to generate immune escape mutants, and comparative sequence analysis with wild-type virus identified potential critical residues. Reverse genetics was used to confirm key sites pivotal for mAb recognition. Structural mapping was performed to localize these sites on the viral capsid.

**Results:**

Ten key sites (K1103, V1215, N1282, F1288, T1291, K2076, E2160, T3059, D3060, E3139) were confirmed to be pivotal for mAb recognition. Structural mapping revealed distinct localization of these sites on the viral capsid: V1215, N1282, F1288, T1291, K2076, E2160, T3059, D3060 at the southern rim; K1103 at the canyon’s northern rim; and E3139 near the two-fold axis.

**Conclusion:**

This study provides the first evidence of multiple conformational neutralizing epitopes on CVA5. These findings define the antigenic basis of CVA5 neutralization and offer a structural framework for vaccine and antibody-based therapeutic development.

## 1 Introduction

Coxsackievirus A5 (CVA5) has long been recognized as a classical pathogen causing hand, foot, and mouth disease (HFMD), herpangina, and acute gastroenteritis in children under 5 years old. Recent epidemiological surveillance reveals its escalating burden globally: CVA5 has been detected in HFMD and related infections across multiple continents, such as South Korea in Asia, Slovenia in Europe, Saudi Arabia in the Middle East, and Tunisia in Africa (1–4). In China, which bears the world’s highest HFMD burden, CVA5 has emerged as a significant non-EVA71 serotype (5). A study from Xiangyang, Hubei Province identified 146 CVA5-positive cases, ranking CVA5 among the six major serotypes causing that epidemic. Phylogenetic and recombination analyses revealed that these CVA5 strains have recombined with CVA2 in the 2A region, potentially contributing to their emergence as a major pathogen (6). Earlier genome analysis of CVA5 isolates from a 2009 outbreak in Shandong Province further confirmed frequent recombination between CVA5 and CVA10 (7).

Developing vaccines represents the primary strategy for preventing enterovirus-associated diseases. A critical challenge in developing effective vaccines against enteroviruses lies in eliciting potent and broadly neutralizing antibodies. Structurally, enteroviruses assemble an icosahedral capsid through proteolytic cleavage of the P1 polyprotein into VP0, VP3, and VP1 to form procapsids. Subsequent RNA packaging triggers VP0 auto-cleavage into VP4 and VP2, forming infectious virions (8). The surface loops of VP1, VP2, and VP3 constitute primary antigenic epitope of enteroviruses (9). Numerous neutralizing mAbs against pathogens including EVA71, CVA10, CVA16, and EV-D68 have been characterized and their cognate epitopes were structurally resolved (9–14). These studies collectively demonstrate that linear epitopes, recognized through sequential amino acid motifs, typically localize to genetically variable surface-exposed loops (15, 16). Conversely, conformational epitopes require intact quaternary structures to maintain antigenic integrity, enabling higher binding specificity and more effective virus neutralization (11–13). Structural analyses further confirm that conformational epitopes dominate functionally critical regions such as the 5-fold symmetry axis, 3-fold symmetry axis, and receptor-binding interfaces (9–11). These structural motifs facilitate high-affinity antibody binding and direct mechanistic interference with viral entry processes (12, 13). For instance, a recent study on CVA16 characterized two neutralizing monoclonal antibodies, 9B5 and 8C4, which target distinct conformational epitopes at the 5-fold and 3-fold protrusions of the viral capsid, respectively. Mechanistically, 9B5 blocks virus attachment by interfering with heparan sulfate binding, while 8C4 inhibits post-attachment uncoating by disrupting SCARB2 interaction, demonstrating that conformational epitopes can mediate neutralization at different stages of viral entry. In contrast, the antigenic architecture of CVA5 remains largely unexplored. In our earlier studies, we established a mAb library against CVA5 and identified a series of mAbs that recognize conserved, linear epitopes (17, 18). Notably, the conformational neutralizing epitopes on CVA5 virions remain unmapped. This knowledge gap directly impedes the rational development of protective vaccines against CVA5 infections.

Conventional epitope mapping approaches exhibit inherent limitations that impede comprehensive antigenic characterization. Peptide-scanning libraries, while effective for linear epitope identification, fail to resolve conformation-dependent antigenicity due to their reliance on discontinuous structural elements (19). Conversely, high-resolution structural techniques (e.g., cryo-EM) are constrained by resource-intensive workflows and low throughput, precluding dynamic profiling of epitope evolution under immune selection pressure (20). To bridge these gaps, in this study, we integrate two synergistic methodologies: mAb-guided escape mutagenesis and reverse genetics-based functional validation. The former mimics natural immune selection by generating neutralization-escape viral mutants, thereby directly correlating residue substitutions with functional epitope disruption (21). The latter employs site-directed mutagenesis of candidate residues to establish causal roles in antibody neutralization, rigorously distinguishing driver mutations from passenger variants (22). This combinatorial strategy uniquely links genetic variation to structural functionality, enabling high-resolution mapping of conformation-dependent neutralizing epitopes and their adaptive landscapes under immune pressure.

## 2 Materials and methods

### 2.1 Viruses

The wild-type CVA5 strain (wt-CVA5, GenBank accession MN663160) was isolated from an anal swab of a HFMD patient in Xiangyang, Hubei Province, China. The isolate underwent three rounds of plaque purification on rhabdomyosarcoma (RD) cells cultured in virus growth medium (Minimum Essential Medium, MEM; Gibco™). Purified virus was amplified through sequential passages in RD cells at a multiplicity of infection (MOI) of 0.01.

### 2.2 Purification of mouse mAbs

Hybridoma cell lines secreting IgG mAbs 3B9, 5E1, secretory IgA mAb 3G1, and IgM mAbs 1H3, 4F5, 1C12, 3H7, 4A5 were expanded in RPMI-1640 medium supplemented with 10% fetal bovine serum. Cells were harvested by centrifugation at 1,000 × g for 5 minutes, washed with sterile PBS, and resuspended to a density of 1×10^6^ cells/mL. Ten female BALB/c mice aged 6–8 weeks per hybridoma line received intraperitoneal injection (i.p.) of 0.5 mL pristane for priming 7–10 days prior. Subsequently, 0.5 mL hybridoma suspension was injected intraperitoneally. Ascites was collected at 7–10 days post injection, incubated at 37 °C for 30 minutes followed by 4 °C overnight, clarified through centrifugation at 500 × g for 30 minutes, and sequentially filtered using 0.22-μm membranes. Antibodies were purified according to subtype-specific methods: Protein A affinity chromatography for mAbs 3B9 and 5E1, Protein L affinity chromatography for mAbs 3G1, 1H3 and 4F5, and two-step 45% saturated ammonium sulfate precipitation for mAbs 1C12, 3H7 and 4A5. Purified mAbs were quantified with Pierce BCA Protein Assay Kit and purity was verified by SDS-PAGE.

### 2.3 Determination of half-maximal effective concentration (EC_50_) by indirect ELISA

Ninety six-well polystyrene plates were coated with purified CVA5 mature virions at 1 μg/mL in 100 μL carbonate-bicarbonate buffer pH 9.6 per well and incubated overnight at 4 °C. After washing with PBST, plates were blocked using 200 μL per well of 1% bovine serum albumin in PBST at 37 °C for 1 hour. Serial twofold dilutions of mAbs starting from 10 μg/mL were added at 100 μL per well and incubated at 37 °C for 1 hour. Following PBST washes, horseradish peroxidase-conjugated secondary antibodies were applied at 1:10–000 dilution: goat anti-mouse IgG for mAbs 3B9 and 5E1, goat anti-mouse IgA for mAb 3G1, and goat anti-mouse IgM for mAbs 1C12, 1H3, 3H7, 4A5 and 4F5. After 1-hour incubation at 37 °C and washing, 100 μL TMB substrate solution was added per well. Plates were incubated at 37 °C for 30 minutes in the dark before terminating reactions with 50 μL per well of 2 M sulfuric acid. Absorbance was measured at 450 nm using a microplate reader. EC_50_ values were calculated through four-parameter logistic curve fitting.

### 2.4 Neutralization assay for half-maximal inhibitory concentration (IC_50_) determination

Neutralization potency of mAbs was quantified via MTT-based cell viability assay. Briefly, serially diluted mAbs (initial concentration 200 μg/mL, twofold dilutions) in 50 μL volumes were mixed with 50 μL wt-CVA5 (100 CCID_50_/50 μL) in 96-well plates. After incubation at 37 °C for 2 hours, the antibody-virus complexes were transferred onto confluent rhabdomyosarcoma (RD) cell monolayers and cultured at 37 °C under 5% CO_2_ for 72 hours. Subsequently, 10 μL MTT reagent was added per well to achieve a final concentration of 0.5 mg/mL, followed by additional 4-hour incubation under identical conditions. The supernatant was carefully aspirated, and 100 μL formazan solubilization solution was added to each well. Plates were agitated on an orbital shaker at 300 rpm for 10 minutes to ensure complete crystal dissolution. Optical density was measured at 570 nm using a microplate reader. The percentage neutralization was calculated as:


$$\text{Percent of neutralization}=(1-\frac{\text{ODsample}-\text{ODcell control}}{\text{ODvirus control}-\text{ODcell control}})\times 100$$

IC_50_ values were derived from four-parameter logistic curve fitting.

### 2.5 Generation of mAb escape mutants

The wt-CVA5 underwent 16 serial passages in rhabdomyosarcoma (RD) cells prior to selection. For escape mutant induction, virus dilutions containing 100 CCID_50_ per 100 μL were mixed with equal volumes of each mAb at 4-fold IC_50_ concentrations. Following mixing by pipetting, antibody-virus complexes were incubated at 37 °C for 2 hours before inoculation onto RD cell monolayers exceeding 80% confluency in 96-well plates. Cultures were maintained at 37 °C under 5% carbon dioxide for 3–5 days until cytopathic effect (CPE) developed. Supernatants from CPE-positive wells were harvested, clarified by centrifugation at 4,000 × g for 10 minutes at 4 °C, and 50 μL aliquots were subsequently subjected to iterative selection cycles at escalating antibody concentrations (first at 8-fold IC_50_ then at 32-fold IC_50_). Viruses demonstrating CPE persistence at the highest antibody concentration were titered by TCID_50_ assay and validated as escape mutants through neutralization resistance testing against both rabbit anti-CVA5 polyclonal serum and the selecting mAb. Finally, antibody-selected viruses underwent three rounds of plaque purification, and sixteen individual clones per antibody were expanded to passage 3 in RD cells for whole-genome sequencing.

### 2.6 Site-directed mutagenesis for generation of recombinant viruses

Nucleotide substitutions within the infectious cDNA clone of wt-CVA5 were introduced using the Mut Express Universal Fast Mutagenesis Kit (Vazyme, Nanjing, China). Specifically designed primers facilitated targeted mutations on plasmid DNA which underwent enzymatic assembly at 37 °C for 30 minutes. Linearized viral cDNA templates were subjected to *in vitro* transcription with T7 RNA polymerase. Resulting genomic RNA was transfected into RD cells using Lipofectamine 2000 (Thermo Fisher Scientific, USA). Rescued viruses were harvested upon complete CPE development. Successful introduction of single amino acid substitutions was confirmed through Sanger sequencing of the entire viral capsid region with no unintended mutations detected.

### 2.7 Structural prediction and epitope mapping of CVA5 capsid proteins

Amino acid sequences of VP1, VP2, VP3, and VP4 from wt-CVA5 were submitted to AlphaFold 3.0 (23) for *de novo* prediction of monomeric and pentameric conformations using default parameters with multiple sequence alignments enabled. The structural outputs were processed through ESPript 3.0 for conserved surface feature annotation including electrostatic potential and hydrophobicity mapping. Final pentameric assemblies were imported into PyMOL version 2.5 for structural analysis where key neutralizing epitopes were mapped through iterative visualization of solvent-accessible surfaces, secondary structure element assignment via DSSP algorithm, and distance-based clustering of antibody escape mutation sites within 5Å radii. Spatial coordinates of functionally validated residues were rendered in molecular graphics with surface topology coloring.

### 2.8 Statistical analysis

The TCID_50_ for each virus in the RD cells was calculated by the Reed-Muench method. All IC_50_ and EC_50_ assays were performed in three independent experiments with two technical replicates per concentration. Data are shown as Mean ± standard deviation (SD). For neutralization comparisons between wild−type and mutant viruses, two−way ANOVA with Tukey’s multiple comparisons was used. A p value less than 0.05 was considered statistically significant. Graphs were generated with Microsoft Excel and GraphPad Prism 10.1.

## 3 Results

### 3.1 Production, characterization, and functional profiling of mouse mAbs

**Figure 1:**
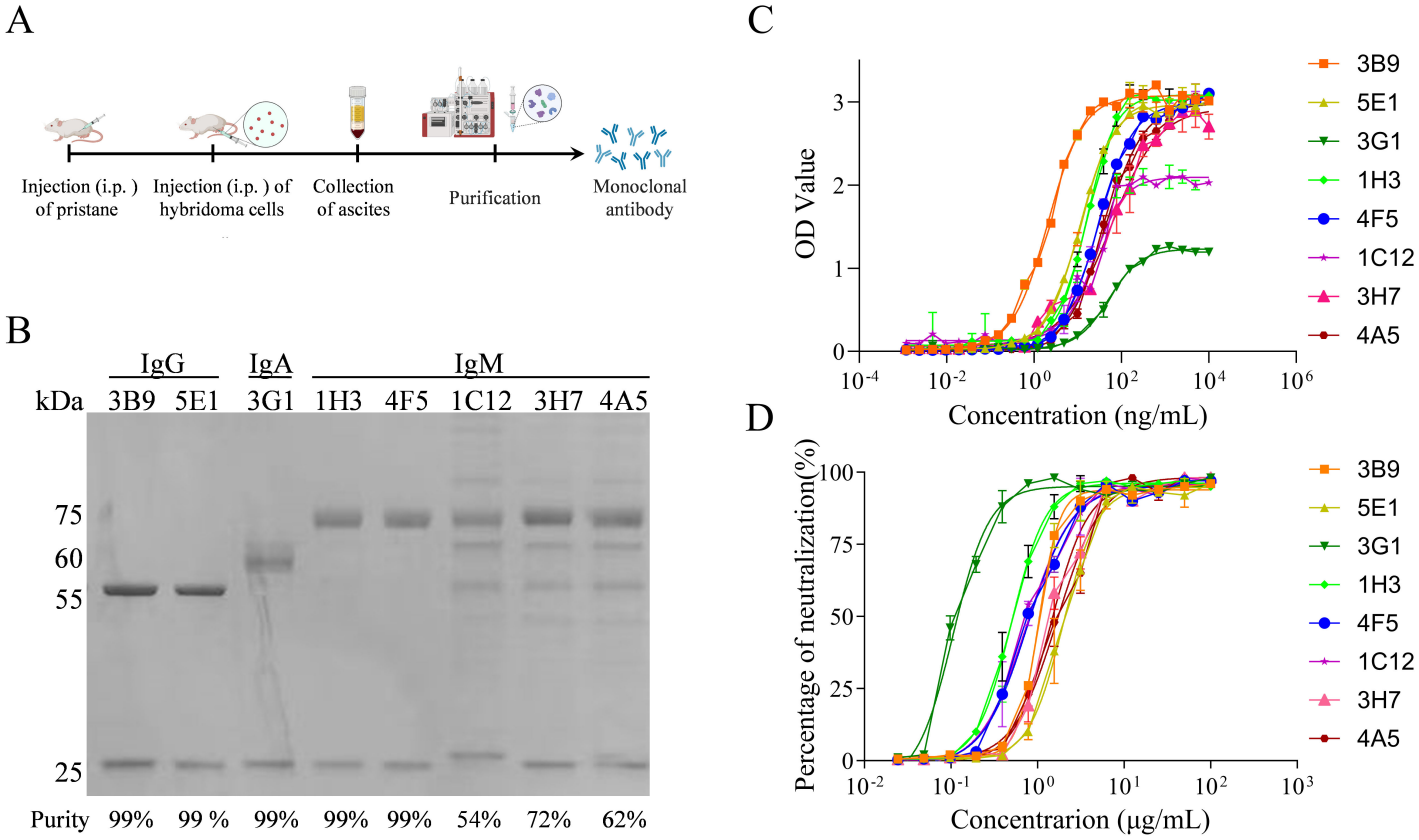
Production, purification, and functional characterization of anti-CVA5 mAbs. **(A)** Workflow for the production and purification of mAbs. **(B)** Reducing SDS-PAGE analysis of the eight purified mAbs. Purity levels, as determined by densitometric analysis, are indicated below each lane. **(C)** Half-maximal effective concentration (EC_50_) values were determined by indirect ELISA using purified CVA5 virions as the capture antigen. **(D)** Half-maximal inhibitory concentration (IC_50_) values were determined using an MTT-based cell viability assay post-infection. Three independent experiments with two technical replicates per concentration. Data are shown as mean ± SD.

Eight hybridoma-derived conformational neutralizing mAbs designated 3B9, 5E1, 3G1, 1H3, 4F5, 1C12, 3H7, and 4A5 were purified from mouse ascites through subtype-specific approaches (**Figure 1A**). IgG-class antibodies 3B9 and 5E1 were purified using Protein A affinity chromatography, whereas IgA-class 3G1 and IgM-class antibodies 1H3 and 4F5 utilized Protein L affinity purification. The IgM-class antibodies 1C12, 3H7, and 4A5 required two-step 45% saturated ammonium sulfate precipitation owing to their inability to bind standard affinity resins. Subsequent SDS-PAGE analysis verified structural integrity with heavy chains resolving at molecular weights characteristic of each isotype: 55 kDa for IgG, 60 kDa for IgA, and 75 kDa for IgM. Light chains consistently migrated at 25 kDa across all subtypes. Quantitative densitometric analysis using Quantity One software demonstrated purity exceeding 99% for affinity-purified antibodies. In contrast, ammonium sulfate-precipitated antibodies exhibited moderate purity levels: 54% for 1C12, 72% for 3H7 and 62% for 4A5 (**Figure 1B**).

To further evaluate the functional properties of these well-characterized antibodies, we next assessed both their antigen-binding affinity and neutralization capacity. Antigen-binding affinity measurements revealed EC_50_ values of 13.3 ng/mL for 3B9, 15.96 ng/mL for 1H3, 23.81 ng/mL for 1C12, 29.10 ng/mL for 4F5, 40.03 ng/mL for 3H7, 42.44 ng/mL for 4A5, 11.14 ng/mL for 5E1, and 51.66 ng/mL for 3G1 (**Figure 1C**). In parallel, quantitative neutralization assays yielded IC_50_ values of 0.52 μg/mL for 1H3, 0.13 μg/mL for 3G1, 0.83 μg/mL for 1C12, 0.85 μg/mL for 4F5, 1.51 μg/mL for 3H7, 1.70 μg/mL for 4A5, 0.96 μg/mL for 3B9, and 1.93 μg/mL for 5E1 (**Figure 1D**). Notably, the IgA-class antibody 3G1 exhibited potent neutralization activity despite its relatively low binding affinity, suggesting a mechanism of action that may depend on conformational epitope recognition. In summary, we successfully purified eight mAbs and comprehensively determined their respective binding affinities and neutralization potencies, establishing a foundation for further epitope characterization.

### 3.2 Identification and characterization of neutralization escape mutants

**Figure 2:**
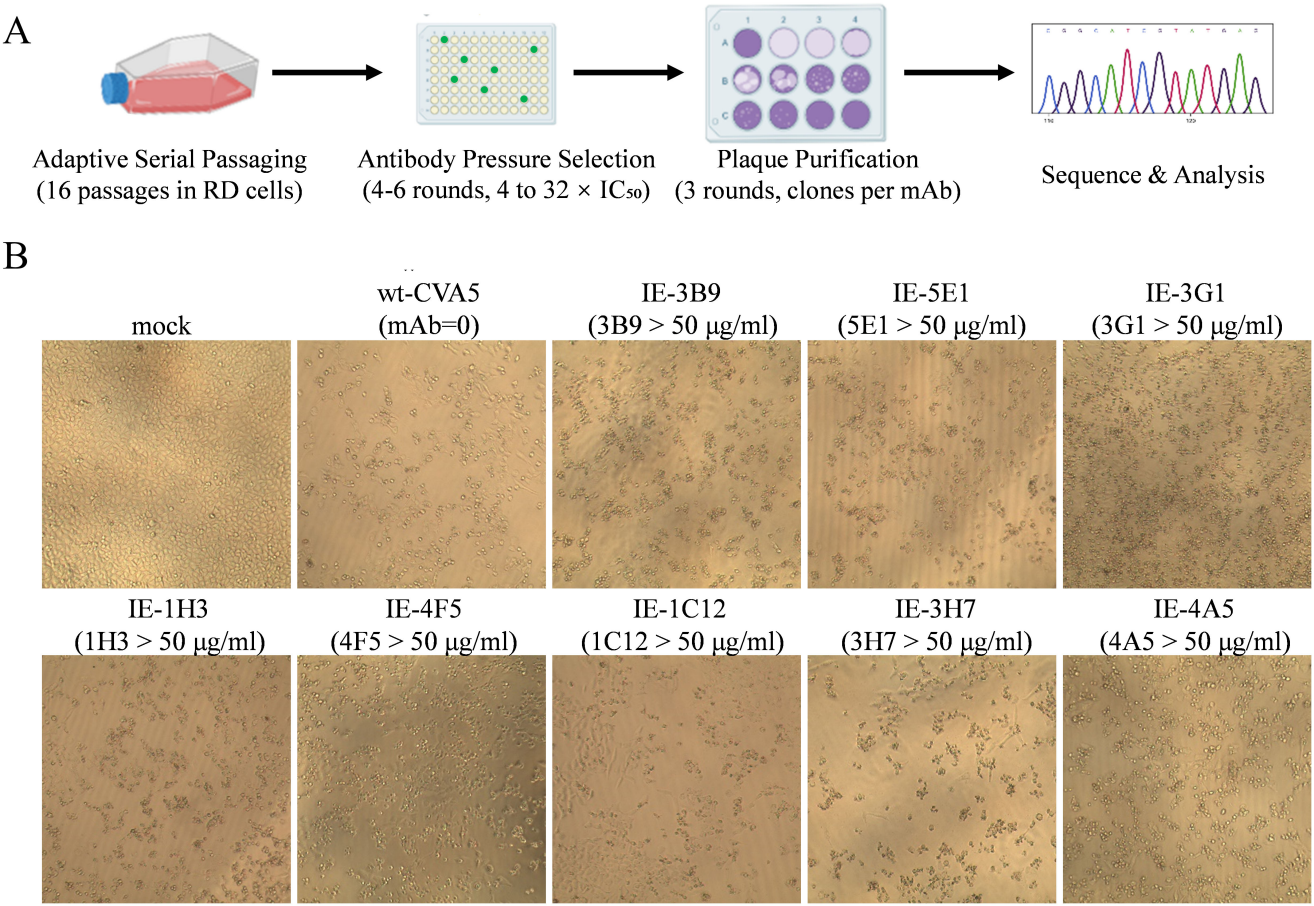
Selection and phenotypic characterization of CVA5 mAb escape mutants. **(A)** Workflow for the selection and identification of escape mutants. **(B)** Representative cytopathic effects (CPE) induced by immune escape variants under high antibody pressure. Escape variants were named IE-mAbs (e.g., IE-3B9, an escape variant selected against mAb 3B9).

To identify the major antigenic epitopes of the conformational neutralizing mAbs, we conducted a comprehensive escape mutant screening study. The overall screening workflow is illustrated in **Figure 2A**. The wild-type CVA5 strain was first pre-adapted through 16 serial passages in RD cells for accumulation of a large variant pool, followed by four to six rounds of selection under escalating pressure in the presence of each mAb. Escape viruses capable of producing distinct CPE in the presence of 50 μg/mL of the corresponding mAb were successfully isolated for all eight antibodies (**Figure 2B**).

To pinpoint the escape mutations related to antibody binding footprints of conformational epitopes, each selected viral population was subjected to plaque purification. Sixteen individual clones per mAb were picked and amplified, all of which maintained the ability to induce CPE under 50 μg/mL antibody pressure. The structural protein-encoding regions of each clone were sequenced and translated, and the resulting amino acid sequences were aligned against the wild-type virus.

Comparative analysis revealed consensus amino acid substitutions common to all escape clones selected by each mAb (**Table 1**). Specifically, escape mutants selected with mAb 3B9 consistently acquired E2160V, E3179K, K1273R, and F1288S. Those selected with 5E1 acquired K2076R, E3179K, and F1288S; escape mutants selected with 3G1 acquired D3060V, E3179K, and F1288S; and those selected with 4F5 acquired T3059D and S1180L. Escape mutants selected with mAb 3H7 acquired H3011R, A1050V, and V1215A, while those selected with 4A5 acquired K2076R, H3011R, and A1050V. Two distinct mutational patterns were identified for mAb 1H3: ten escape clones acquired E3139K and N1282Y, while the other six acquired E3139K, K1103E, and T1291A. All escape clones selected with mAb 1C12 acquired mutations at F1288, with twelve clones acquiring F1288S and four acquiring F1288L. These identified amino acid residues represent potential determinants of neutralization escape and are likely footprints for the recognition of conformational epitopes by the respective mAbs.

**Table 1:** Antibody-associated immune escape mutations*.

Antibody	Shared mutation sites
3B9	E2160V, E3179K, K1273R, F1288S
5E1	K2076R, E3179K, F1288S
3G1	D3060V, E3179K, F1288S
1H3	E3139K + N1282Y (10) or E3139K + K1103E + T1291A (6)
4F5	T3059D, S1180L
1C12	F1288S (12) or F1288L (4)
3H7	H3011R, A1050V, V1215A
4A5	K2076R, H3011R, A1050V

*The table lists the shared immune escape mutation sites identified across 16 monoclonal escape variants for each antibody. The plus symbol (+) denotes mutation combinations found within the same viral variant. The notation “or” indicates distinct major mutational profiles. Numbers in parentheses represent the count of plaque-purified clones carrying the corresponding mutation(s).

### 3.3 Validation of key neutralizing determinants via reverse genetics

To definitively identify the critical amino acid residues responsible for neutralization escape, we employed a reverse genetics system to generate a series of recombinant CVA5 viruses bearing individual or combined amino acid substitutions. All rescued recombinant viruses induced cytopathic effect (CPE) comparable to rescued wild-type CVA5 (rwt-CVA5) and achieved titers of at least 10^6^ CCID_50_ at passage 2, indicating that the introduced mutations did not severely compromise viral replication. The neutralizing activity of each mAb against these mutants was then systematically assessed (**Table 2**). For mAb 3B9, escape mutants had consistently acquired E2160V, E3179K, K1273R, and F1288S. However, neutralization assays against the panel of recombinant viruses revealed that the single E2160V substitution was alone sufficient to confer complete resistance to 3B9 neutralization. In contrast, the E3179K, K1273R, and F1288S mutations individually did not significantly alter viral sensitivity to 3B9, unequivocally identifying E2160 as the critical determinant for this antibody. This same approach precisely mapped the primary neutralizing determinants for other mAbs: a single K2076R substitution conferred complete escape from 5E1; T3059D from 4F5; F1288S/L from 1C12; V1215A from 3H7; and K2076R from 4A5. A distinct pattern was observed for mAbs 3G1 and 1H3. For 3G1, only the double mutant (D3060V+F1288S) exhibited complete resistance, defining both D3060 and F1288 as critical cooperative residues. For 1H3, complete escape required combined mutations, as evidenced by two distinct resistant genotypes: E3139K+N1282Y and E3139K+K1103E+T1291A, identifying E3139, N1282, K1103, and T1291 as key residues (**Table 3**). Collectively, the reverse genetics system enabled high-resolution mapping of the CVA5 antigenic landscape by definitively identifying single and combinatorial amino acid residues that are essential for antibody-mediated neutralization.

**Table 2:** Neutralization of recombinant viruses engineered with escape mutations.

Antibody	Strain	Neutralization concentration (μg/ml)
3B9	rwt-CVA5	0.894
	rCVA5-E2160V*	**≥100** ^*^
	rCVA5-E3179K	0.894
	rCVA5-K1273R	1.341
	rCVA5-F1288S	0.894
5E1	rwt-CVA5	1.748
	**rCVA5-K2076R**	**≥100** ^*^
	rCVA5-E3179K	1.852
	rCVA5-F1288S	1.852
3G1	rwt-CVA5	0.121
	rCVA5-D3060V	7.744^*^
	rCVA5-E3179K	0.121
	rCVA5-F1288S	0.121
	**rCVA5-D3060V + F1288S**	**≥100** ^*^
	rCVA5-D3060V + E3179K	7.744^*^
	rCVA5-E3179K + F1288S	0.181
1H3	rwt-CVA5	0.561
	rCVA5-E3139K	0.841
	rCVA5-K1103E	0.561
	rCVA5-N1282Y	0.561
	rCVA5-T1291A	0.561
	**rCVA5-E3139K + N1282Y**	**≥100** ^*^
	rCVA5-E3139K + T1291A	0.841
	rCVA5-E3139K + K1103E	0.561
	rCVA5-K1103E + T1291A	0.841
	**rCVA5-E3139K + K1103E + T1291A**	**≥100** ^*^
4F5	rwt-CVA5	0.801
	**rCVA5-T3059D**	**≥100** ^*^
	rCVA5-S1180L	0.801
1C12	rwt-CVA5	0.813
	rCVA5-H3011R	0.813
	rCVA5-A1050V	3.252
	**rCVA5-F1288S**	**≥100** ^*^
	**rCVA5-F1288L**	**≥100** ^*^
3H7	rwt-CVA5	1.095
	rCVA5-H3011R	1.095
	rCVA5-A1050V	1.095
	**rCVA5-V1215A**	**≥100** ^*^
4A5	rwt-CVA5	1.748
	**rCVA5-K2076R**	**≥100** ^*^
	rCVA5-H3011R	1.748
	rCVA5-A1050V	2.622

Values in bold indicate complete escape (neutralization concentration ≥ 100 μg/mL).

*Neutralization concentration with statistically significant difference from wt-CVA5.

**Table 3:** Key neutralizing amino acid residues of CVA5 for mAbs.

Antibody	Critical residues
3B9	E2160
5E1	K2076
3G1	D3060, F1288
1H3	E3139, N1282, K1103, T1291
4F5	T3059
1C12	F1288
3H7	V1215
4A5	K2076

### 3.4 Structural mapping of key conformational neutralizing epitopes on CVA5

**Figure 3:**
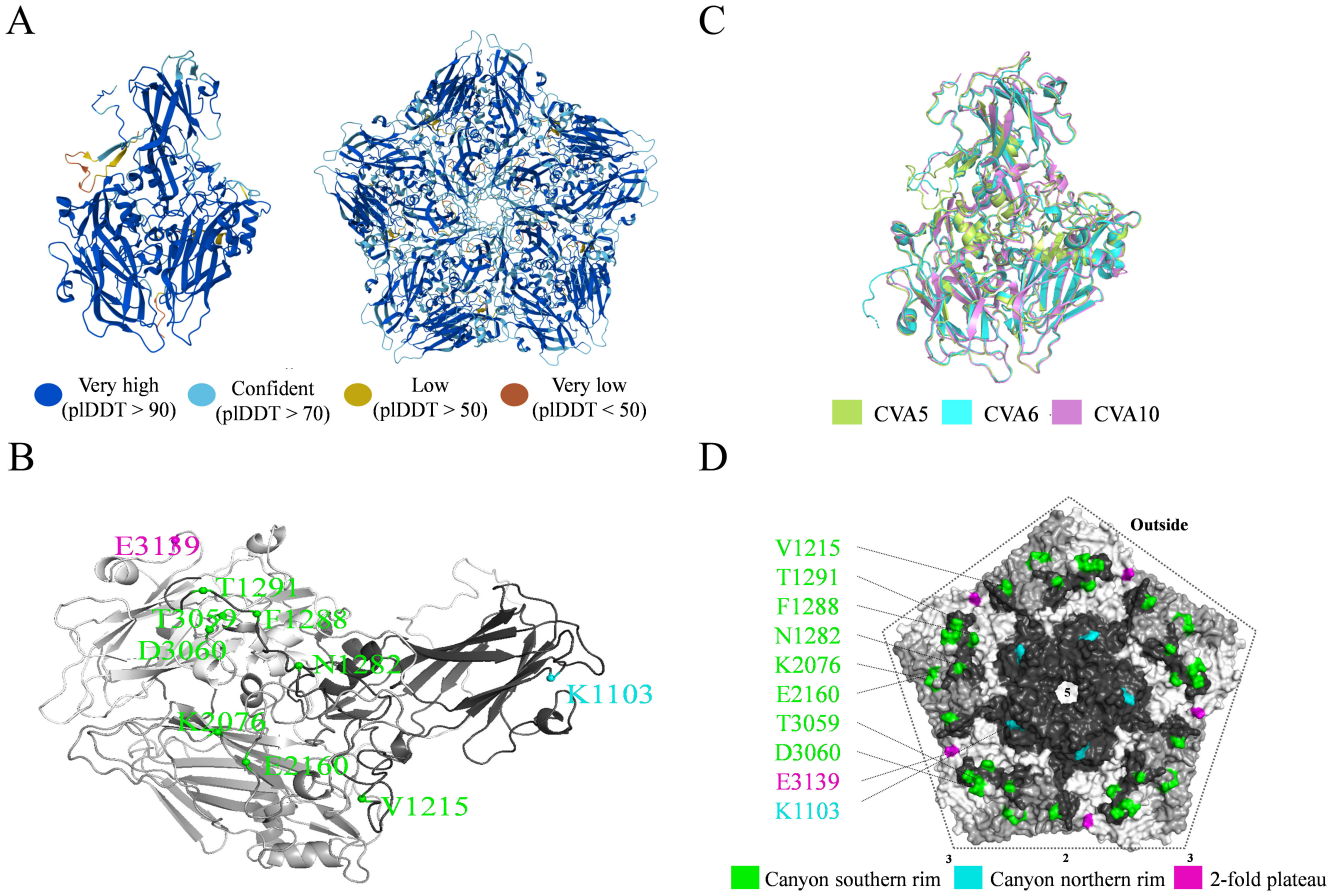
Structural prediction and high-resolution mapping of conformational neutralizing epitopes on CVA5. **(A)** Predicted structures of a CVA5 capsid protomer (left) and pentamer (right) generated by AlphaFold 3.0. The model is colored by per-residue confidence (pLDDT score), with blue indicating high confidence and orange/red indicating lower confidence. **(B)** Structural alignment of the predicted CVA5 protomer (green) with experimentally determined structures of CVA6 (cyan, PDB: 7QW9) and CVA10 (purple, PDB:6SMG). Precise mapping of critical neutralizing residues identified through escape mutagenesis onto the **(C)** protomer and **(D)** pentamer structures. Residues are colored according to their topological location on the viral capsid (green, canyon southern rim; cyan, canyon northern rim; purple, 2-fold plateau).

The capsid of CVA5 is composed of sixty copies each of the four structural proteins VP1 to VP4, which first assemble into protomers. Five protomers form a pentameric unit, and twelve such pentamers encapsidate the viral genome to form an infectious virion. To map the spatial locations of the identified neutralizing epitopes, the amino acid sequence of the wt-CVA5 strain was used to predict both monomeric and pentameric structures using AlphaFold 3.0 (**Figure 3A**). The prediction yielded high-confidence models. The pLDDT score, reflecting per-residue confidence, was ≥ 90 for the vast majority of residues, with all epitope-associated residues exhibiting pLDDT > 85, indicating very high accuracy (23). The predicted CVA5 monomer achieved an interface predicted TM-score (ipTM) of 0.83 and a predicted TM-score (pTM) of 0.86. The pentameric complex prediction was also of high quality, with an ipTM of 0.80 and a pTM of 0.81. These scores (ipTM > 0.8 and pTM > 0.5) signify that the predicted folds for both the monomer and the pentamer are of high quality and highly similar to the true native structures (24). The predicted monomer structure exhibited high similarity to known enterovirus structures, with root-mean-square deviation (RMSD) values of 0.480 and 0.474 when aligned against CVA6 and CVA10 structures from the PDB database, respectively (RMSD < 0.6 indicates high structural similarity) (**Figure 3B**) (25, 26).

This reliable structural model enabled the precise mapping of critical neutralizing residues: A1050 was localized to the VP1 AB loop; K1103 to the VP1 BC loop; V1215 to the VP1 GH loop; N1282, F1288, and T1291 to the C-terminal region; K2076 to the VP2 BC loop; E2160 to the VP2 EF loop; T3059 and D3060 to the VP3 AB loop; and E3139 to a VP3 loop (**Figure 3C**). Projection of these residues onto the high-confidence pentameric structure confirmed that all are situated on the exposed viral surface. Notably, V1215, T1291, F1288, N1282, K2076, E2160, T3059 and D3060 clustered predominantly along the south rim of the canyon, a region pivotal for receptor engagement (**Figure 3D**). In contrast, E3139 was positioned on the north rim of the canyon, while K1103 was located near the two-fold symmetry axis. This high-resolution structural map reveals that the identified conformational neutralizing epitopes of CVA5 are organized across exposed loops and termini of the capsid proteins, with a major cluster of key residues at the biologically critical south rim of the canyon, providing a structural basis for understanding antibody-mediated neutralization. However, these structural predictions are derived solely from computational models, and experimental validation (e.g., by cryo-EM) remains necessary for further verification.

## 4 Discussion

CVA5 has emerged as a significant pathogen responsible for severe hand, foot, and mouth disease, yet its antigenic landscape remained largely unexplored. Our study provides the first comprehensive mapping of the conformational neutralizing epitopes on the CVA5 capsid, revealing a complex antigenic architecture that underpins antibody-mediated neutralization and escape. By integrating mAb escape mutagenesis with reverse genetics validation, we precisely defined ten key amino acid residues (K1103, V1215, N1282, F1288, T1291, K2076, E2160, T3059, D3060, E3139) that are critical for neutralization by eight distinct conformational mAbs. Importantly, we delineated two distinct mechanisms of neutralization escape: for most mAbs, a single amino acid substitution was sufficient to confer complete resistance, whereas for others (notably 3G1 and 1H3), complete escape required cooperative changes at multiple sites. Collectively, these findings fundamentally advance our understanding of CVA5 antigenicity and provide a robust structural framework for the rational design of vaccines and therapeutics targeting this re-emerging virus.

The credibility of our mapped epitopes is strongly reinforced by their striking evolutionary conservation and functional congruence with antigenic sites in other enteroviruses. Although none of these specific residues have been previously reported in CVA5, sequence and structural alignments using ESPript 3.0 with CVA6, CVA10, CVA16, and EVA71 (**Supplementary Figures 1**–**3**) reveal that the relative positions of these sites are established determinants of neutralization. For instance, N1282, has been documented as part of a conformational epitope in CVA10 in conjunction with spatially adjacent residues Y2162 and D1289 (27). The corresponding residue in EVA71 has likewise been identified as participating in conformational epitope formation (9, 28). Similarly, T1291, has been consistently reported as a key component of neutralization epitopes in EVA71 and CVA16 (28, 29). The residues K1103, V1215, E2160, T3059, E3139 have likewise been implicated in forming conformational epitopes in CVA16 and EVA71, and D3060 has been identified in both CVA10 and EVA71 (9, 27–31). This cross-serotype conservation underscores the high confidence of our identified epitopes and validates the rationale of using epitope mapping in one enterovirus to inform studies on others.

Notably, our study reveals two novel antigenic sites without precedent in HFMD-associated enteroviruses: K2076 and the F1288 region. The most functionally insight stems from the spatial relationship between these neutralizing epitopes and the known receptor-binding site. CVA5 utilizes the KREMEN1 receptor for cell entry (32). Our analysis shows that two of our identified residues, F1288 and T1291, directly overlap with amino acid positions reported to make intimate contact (≤4 Å) with KREMEN1 in CVA10. Furthermore, three other residues—V1215, T3059, and D3060—are located in close proximity to the KREMEN1 binding footprint (33, 34). All five of these mechanistically critical residues are situated on the south rim of the canyon, which is the primary interface for KREMEN1 binding. Enterovirus infection begins with attachment of the viral capsid to cell surface receptors, triggering conformational rearrangements that lead to genome uncoating and release into the cytoplasm. Neutralizing antibodies can interfere with this process at multiple levels: they may block receptor engagement by sterically masking the receptor-binding site, prevent receptor-induced conformational changes required for uncoating, or even trigger premature genome release (11, 28). This compelling spatial coincidence strongly suggests that antibodies targeting these five specific sites likely mediate potent neutralization by sterically hindering or allosterically disrupting the critical virus-receptor interaction, thereby directly preventing the initiation of infection. We emphasize that this receptor-blocking hypothesis is based solely on spatial proximity to the known KREMEN1 binding footprint in CVA10 and has not been experimentally validated. Such receptor-blocking mechanisms have been well documented for other enteroviruses, where antibodies targeting canyon-proximal epitopes inhibit viral entry at the attachment or post-attachment stages (13, 29).

Our study not only mapped the epitopes but also proposed two distinct mechanistic pathways of neutralization escape. For six of the eight mAbs (3B9, 5E1, 4F5, 1C12, 3H7, 4A5), a single amino acid substitution was sufficient to confer complete immune escape. This indicates that these mAbs recognize discrete, focused antigenic sites where the binding affinity and neutralization activity are critically dependent on a single “anchor” residue. The disruption of a key interaction at this specific site is enough to abrogate antibody neutralizing activity entirely. This high specificity often correlates with exceptionally potent neutralization but also renders the antibody highly vulnerable to single-point escape mutants, a potential limitation for monotherapeutic applications (14, 35). In contrast, complete escape from mAbs 3G1 and 1H3 required cooperative changes at multiple sites. For 3G1, both D3060V and F1288S mutations were necessary, suggesting that it recognizes a complex, quaternary epitope where the antibody binding interface spans two distinct residues. Neither single mutation likely induces a sufficient conformational change or steric clash to prevent binding, but the double mutation disrupts the epitope’s integrity. The most complex pattern was observed for 1H3, where the virus escaped via two distinct genetic pathways (E3139K+N1282Y or E3139K+K1103E+T1291A). This demonstrates that the 1H3 epitope is particularly extensive and structurally constrained, making it difficult for the virus to escape without a fitness cost. The requirement for multiple, simultaneous mutations to escape such antibodies presents a significantly higher genetic barrier for the virus (36). This makes antibodies like 1H3 and 3G1 highly attractive candidates for therapeutic development, as their targets are less likely to be circumvented by a single viral mutation, potentially leading to more durable treatment responses and higher resilience against viral escape.

In conclusion, our study provides the first high-resolution functional and structural map of conformational neutralizing epitopes for CVA5, revealing key insights into its antigenic architecture. The identification of both conserved and novel epitopes, along with their precise mechanistic roles in receptor blocking and virus neutralization, offers a robust foundation for rational countermeasure design. Owing to the difficulty of obtaining sufficient purified mutant virions, direct binding assays were not performed; thus, the identified residues are functional neutralization determinants rather than definitive physical binding footprints. Future efforts should focus on obtaining cryo-EM structures of complexes formed between Fab fragments of antibodies and viral capsids to visualize these interactions at atomic resolution and evaluating the *in vivo* efficacy of selected antibodies or epitope-based immunogens. Notably, the moderate purity of the IgM antibodies does not affect our epitope mapping conclusions, as the neutralization concentration (≥ 100 μg/mL) was approximately 100-fold higher than that for the wild-type virus, rendering any potential impact of incomplete purity negligible. Despite the limitation of relying on predictive modeling, this work establishes a critical framework for developing CVA5-specific vaccines and antibody-based therapeutics, and underscores the value of cross-serotypic epitope mapping for anticipating antigenic trends among enteroviruses.

## Data Availability

The original contributions presented in the study are included in the article/**Supplementary Material**. Further inquiries can be directed to the corresponding authors.
