# Supplementary material for: Identification of key amino acids defining conformational neutralizing epitopes of coxsackievirus A5 using monoclonal antibody escape mutants
Source: Front Immunol. 2026 Apr 29;17:1837533. doi: 10.3389/fimmu.2026.1837533 (PMC13167979; doi:10.3389/fimmu.2026.1837533)
Supplement: Supplementary file 1 [file DataSheet1.pdf]

Supplemental material

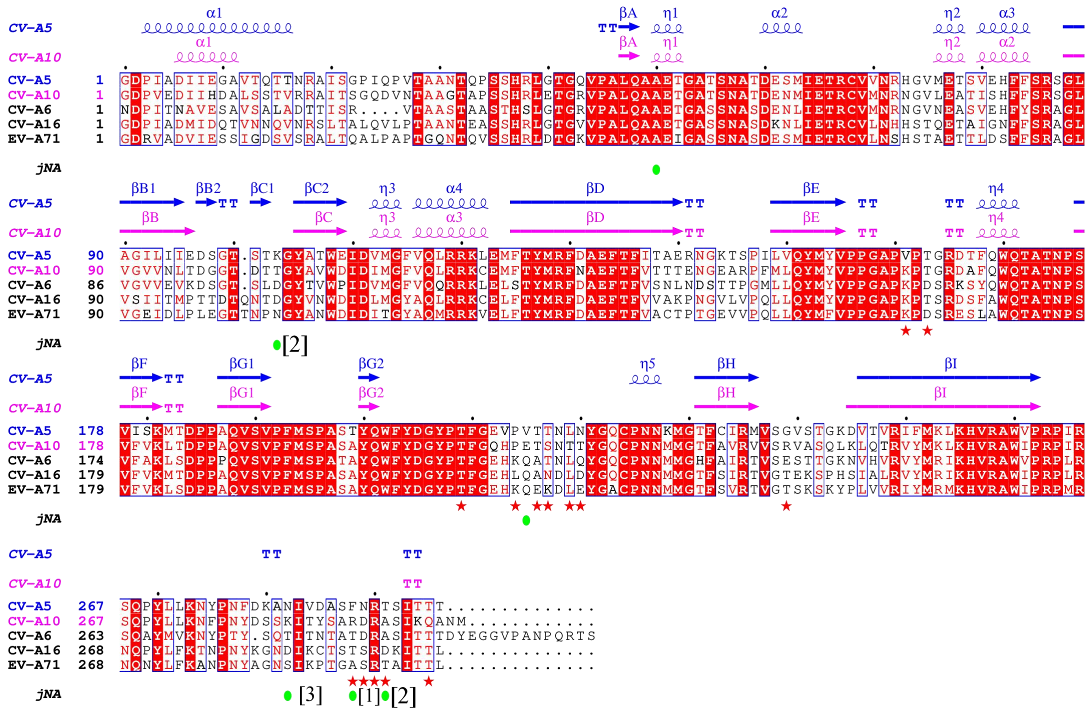

Figure S1. Sequence alignment of the VP1 amino acid secondary structures of representative strains of enterovirus A species.

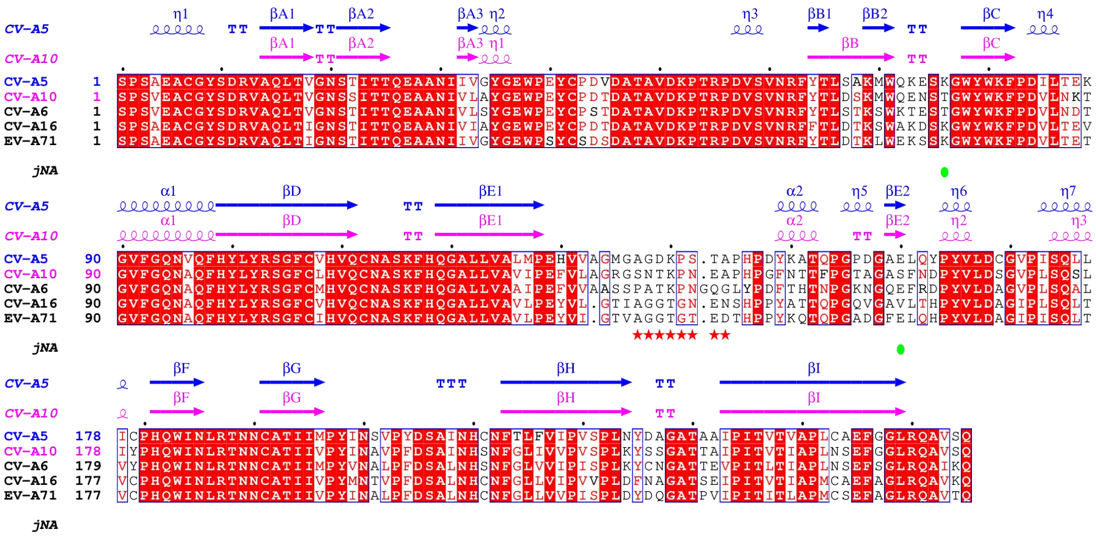

Figure S2. Sequence alignment of the VP2 amino acid secondary structures of representative strains of enterovirus A species.

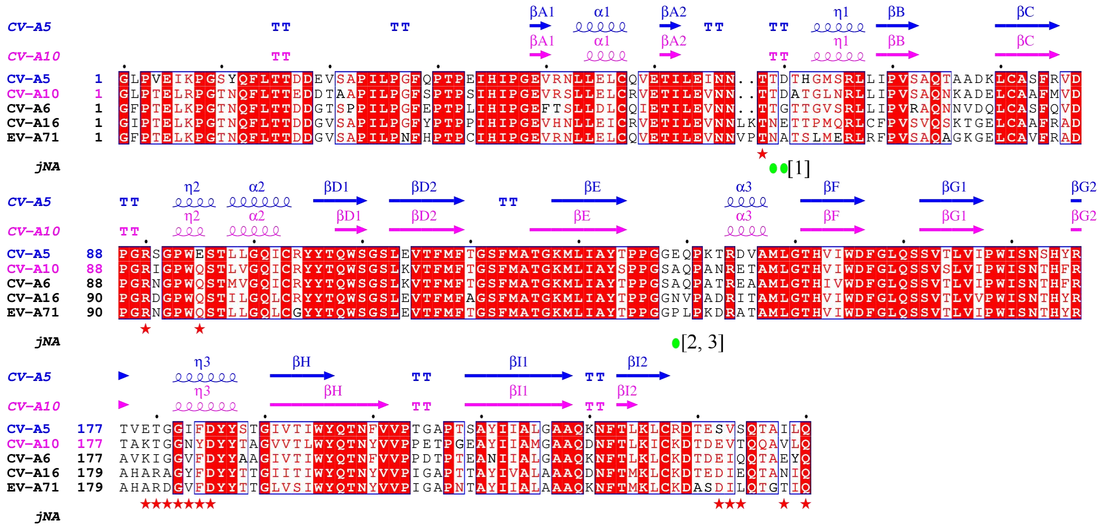

Figure S3. Sequence alignment of the VP3 amino acid secondary structures of representative strains of enterovirus A species.

The alignment includes five enteroviruses: CVA5 (GenBank: MN663160.1), CVA10 (PDB: 6SMG), CVA6 (GenBank: MW410845), CVA16 (GenBank: WIL60470.1), and EVA71 (GenBank: XAM23997.1). Secondary structure elements of CVA5 and CVA10 are displayed above the sequences, colored in blue (CVA5) and pink (CVA10). α-helices, η-helices (3_{10}-helices), and β-strands are denoted by α, η, and β, respectively. “TT” indicates a β-turn, and “TTT” indicates an α-turn. The superscript “★” marks the KREMEN1 contact residues on CVA10 (distance ≤4 Å). “●” indicates critical neutralization sites on CVA5. “● [1] ● [2] ● [3]” marks multiple residue combinations that constitute key antigenic epitopes: ● [1] F1288+D3060; ● [2] K1103+T1291+E3139; ● [3] N1282+E3139.
